# Predictors of the Willingness to Undergo Rhinoplasty: A Psychosocial and Demographic Study From Saudi Arabia

**DOI:** 10.7759/cureus.99913

**Published:** 2025-12-23

**Authors:** Nisreen Albouq, Abdulrahman Alosaimi, Ibrahim A Tawfiq, Naeem Makhdoom, Sulaiman A Althobaiti

**Affiliations:** 1 Otolaryngology - Head and Neck Surgery, Ohud Hospital, Medinah, SAU; 2 General Practice, Taibah University, Medinah, SAU; 3 Otolaryngology - Head and Neck Surgery, Taibah University, Medinah, SAU; 4 Otolaryngology - Head and Neck Surgery, King Abdulaziz Specialist Hospital, Taif, SAU

**Keywords:** aesthetic predictors, facial plastic surgery, nasal satisfaction, rhinoplasty outcome evaluation, rhinoplasty surgery, surgical willingness

## Abstract

Background

Rhinoplasty is increasingly being performed for aesthetic enhancement and functional improvement. However, the decision to undergo rhinoplasty involves physical, psychological, and social factors. This study investigated which nasal features and demographic factors predict the willingness to undergo rhinoplasty.

Methodology

A cross-sectional online survey was conducted using a structured questionnaire administered to patients presenting to ENT clinics with deviated nasal septum or external nasal deformity and via social media platforms in Saudi Arabia to assess demographic characteristics, nasal satisfaction, and willingness to undergo rhinoplasty. Data from 675 eligible participants were analyzed using descriptive statistics, t-tests, analysis of variance, and ordinal logistic regression to identify significant predictors.

Results

Strong dissatisfaction with overall nasal shape significantly increased the willingness to undergo rhinoplasty (p < 0.001). In contrast, dissatisfaction with specific features such as nostril size (p = 0.009) and nose height (p = 0.042) decreased surgical willingness. Females reported lower satisfaction than males across multiple features, though this did not translate into significant differences in surgical motivation (p = 0.064). Educational level significantly influenced rhinoplasty motivations (p < 0.001). In contrast, age, nationality, and employment status did not significantly affect satisfaction or motivation.

Conclusions

The decision to pursue rhinoplasty is personal and influenced by physical, psychological, and social factors. General dissatisfaction with the shape of the nose was the strongest predictor of surgical willingness. However, dissatisfaction with specific aspects of the nose negatively correlated with surgical willingness.

## Introduction

Rhinoplasty reconstructs the nose for aesthetic or functional improvement by changing its size, shape, or proportions [1]. Rhinoplasty is a common facial plastic surgical procedure for both aesthetic and functional improvements [2]. It effectively addresses breathing difficulties caused by structural abnormalities while simultaneously improving patients’ facial aesthetics [1,3].

The number of people undergoing facial surgery has been rising worldwide [4]. This trend reflects a sociocultural shift in aesthetic views, greater access to cosmetic procedures, and technological advances improving outcomes and reducing recovery times [4-6]. In Saudi Arabia, rhinoplasty procedures have recently increased substantially, now representing 30% of all cosmetic surgeries in the country [7]. With advances in rhinoplasty, it is important for practitioners and researchers to understand the intricate factors underlying the patient’s choice to undergo rhinoplasty.

The nose is key to facial beauty, self-concept, and social interaction. Social psychology has shown that the shape influences first impressions, beauty, and personality judgments [8,9]. This makes rhinoplasty a personal decision extending beyond aesthetics to include psychological adjustment, social function, and self-concept [10]. The patients’ motivation to pursue rhinoplasty reflects the complexity associated with aesthetics and functional problems.

The decision to undergo rhinoplasty involves physical, psychological, and social factors. However, the predictive connections between nasal features, demographics, and the decision are inconclusive. Understanding this predictive framework, beyond surgical outcomes or satisfaction, is vital in consultation and planning [11].

Current clinical approaches often rely on the surgeon’s experience and judgment rather than evidence-based models that could better align patient expectations with outcomes. Additionally, while demographic differences in cosmetic surgery utilization have been observed, the specific ways age, gender, and education influence nasal satisfaction and surgical willingness remain under-reported [12,13].

This study aimed to identify the predictors of surgical interest in undergoing rhinoplasty across diverse populations of Saudi Arabia. In addition, it aimed to explore which nasal features and demographic factors, such as gender, age, and education, are most strongly associated with the decision to consider rhinoplasty. The study also aimed to examine differences in nasal satisfaction between genders or age groups, and how motivations for rhinoplasty (aesthetic versus functional) vary across demographic profiles. Incorporating a predictive model linking specific nasal features to rhinoplasty interest is essential to improve patient-centered care in facial plastic surgery.

## Materials and methods

This cross-sectional study was conducted between August 2024 and December 2024 to investigate the predictors of willingness to undergo rhinoplasty and to assess demographic differences in nasal satisfaction among the general population in Saudi Arabia. Ethical approval was obtained from the Medina Health Cluster (approval number: 22-058). All participants provided electronic informed consent before participation. Responses were anonymized to ensure confidentiality and compliance with ethical research standards.

A new questionnaire was developed specifically for this study to assess demographic characteristics (age, gender, education level, employment status, and nationality), levels of satisfaction with various nasal features, and motivations and willingness to undergo rhinoplasty. The survey instrument was reviewed by ENT specialists and pilot-tested on a small group to ensure clarity and relevance. Satisfaction with nasal features was measured using a five-point Likert scale ranging from “Strongly Dissatisfied” to “Strongly Satisfied.” Motivations for rhinoplasty were categorized as aesthetic, functional, or both.

The questionnaire was created in Google Forms and distributed electronically via social media platforms, including X, Instagram, and WhatsApp, to ensure a broad and diverse reach within the Kingdom of Saudi Arabia. Moreover, it was distributed among patients presenting to ENT clinics with deviated nasal septum or external nasal deformities. Responses were automatically recorded for accuracy and data integrity. The form was made available in Arabic and English to accommodate language preferences.

Adults aged 18 years and older residing in Saudi Arabia who could provide informed consent and complete the questionnaire were eligible to participate. Individuals under 18 years of age, living outside Saudi Arabia, or submitting incomplete responses were excluded. A total of 675 complete and eligible responses were included in the final analysis.

The questionnaire was subjected to reliability testing via a pilot testing phase before data collection to establish both validity and reliability. Internal consistency was assessed using Cronbach’s alpha, with all domains demonstrating coefficients above 0.70, indicating acceptable reliability. Content validity was further evaluated by a panel of otolaryngology specialists and research methodologists, who examined each item for clarity, relevance, and cultural appropriateness.

The sample size was determined based on a medium effect size, a 95% confidence level, and 80% statistical power. This calculation indicated a minimum required sample of 385 participants. To increase the precision of the results and account for potential exclusions, a total of 675 individuals were ultimately included in the analysis. A convenience sampling method was employed, recruiting participants through outpatient clinics and online platforms. To ensure the integrity of the dataset, duplicate entries were identified using unique digital identifiers (email/IP address) and removed. Incomplete or inconsistent responses were also excluded to retain only valid and reliable data for analysis.

All data were analyzed using SPSS Statistics version 30 (IBM Corp., Armonk, NY, USA). Descriptive statistics were used to summarize participant characteristics and satisfaction ratings. Ordinal logistic regression was employed to identify predictors of willingness to undergo rhinoplasty, and model fit was assessed using the chi-square test. Gender differences in satisfaction were analyzed using independent samples t-tests, while age-related variations were evaluated through one-way analysis of variance (ANOVA). Associations between motivations for rhinoplasty and demographic variables (age, gender, nationality, education, and employment status) were tested using Pearson’s chi-square and likelihood ratio tests.

A significance level of p < 0.05 was applied to all statistical tests. Both significant and non-significant findings are reported to ensure a comprehensive and transparent interpretation. This multi-method analytical approach allowed for a robust examination of the anatomical, psychological, and demographic factors influencing rhinoplasty interest.

Before conducting inferential analyses, all relevant statistical assumptions were examined. Normality of continuous variables was assessed using the Shapiro-Wilk test, supplemented by visual inspection of histograms and Q-Q plots. Homogeneity of variances was evaluated through Levene’s test before performing independent-samples t-tests, and the same procedure was applied before one-way ANOVA analyses. When assumptions were not fully met, appropriate statistical adjustments were applied to ensure the robustness of the findings.

To promote clarity and transparency, results are presented using a consistent statistical reporting format. Independent-samples t-tests are reported with corresponding t values, degrees of freedom, and p-values. One-way ANOVA results are presented with F statistics, degrees of freedom, and p-values. Ordinal logistic regression outputs include regression coefficients, standard errors, significance levels, and 95% confidence intervals (CIs). A significance level of p < 0.05 was applied across all analyses.

## Results

A total of 675 responses were analyzed. The results focus on the predictors of willingness to undergo rhinoplasty, based on satisfaction with nasal features and demographics. Descriptive statistics are followed by ordinal logistic regression to identify significant predictors. Differences by gender, age, and education level are also reported.

Predictors of willingness to undergo rhinoplasty

To identify key predictors of willingness for rhinoplasty, an ordinal logistic regression model was constructed and analyzed. The primary objective was to determine which nasal features and demographics significantly influenced this willingness. A detailed analysis of predictors (Table 1) revealed several statistically significant associations with willingness to undergo rhinoplasty, notably satisfaction with nasal features. The ordinal logistic regression model showed a statistically significant fit. The chi-square test yielded a p-value of less than 0.001 (chi-square = 144.415, df = 44), indicating that the predictors included in the model collectively provide valuable information for explaining the variation observed in the dependent variable, which is the willingness to undergo rhinoplasty. This robust model fit suggested that the chosen variables are pertinent in understanding the factors contributing to an individual’s inclination toward rhinoplasty (Table 2).

**Table 1:** Detailed analysis of predictors of willingness to undergo rhinoplasty.

	Estimate	Standard error	Significance	95% confidence interval	
				Lower bound	Upper bound
What is your satisfaction with the size of the nostril? = Dissatisfied	-0.954	0.367	0.009	-1.674	-0.234
What is your satisfaction with the size of the nostril? = Neutral	0.057	0.342	0.868	-0.613	0.727
What is your satisfaction with the size of the nostril? = Satisfied	0.257	0.270	0.341	-0.272	0.786
What is your satisfaction with the size of the nostril? = Strongly dissatisfied	-0.707	0.578	0.221	-1.840	0.426
What is your satisfaction with the size of the nostril? = Strongly satisfied	0	-	-	-	-
What is your satisfaction with the height of the nose = Dissatisfied	-0.837	0.412	0.042	-1.645	-0.029
What is your satisfaction with the height of the nose = Neutral	-0.325	0.342	0.342	-0.995	0.345
What is your satisfaction with the height of the nose = Satisfied	-0.479	0.286	0.094	-1.039	0.081
What is your satisfaction with the height of the nose = Strongly dissatisfied	0.290	0.627	0.644	-0.938	1.518
What is your satisfaction with the height of the nose = Strongly satisfied	0	-	-	-	-
What is your satisfaction with the shape of the nose in general = Dissatisfied	1.947	0.417	<0.001	1.129	2.764
What is your satisfaction with the shape of the nose in general? = Neutral	-0.194	0.356	0.585	-0.891	0.503
What is your satisfaction with the shape of the nose in general? = Satisfied	-0.219	0.281	0.436	-0.769	0.332
What is your satisfaction with the shape of the nose in general? = Strongly dissatisfied	3.041	0.609	<0.001	1.847	4.234
What is your satisfaction with the shape of the nose in general? = Strongly satisfied	0	-	-	-	-

**Table 2:** Ordinal logistic regression model fits.

Model	-2 log likelihood	Chi-square	df	Significance
Intercept only	946.233	144.415	44	<0.001
Final	801.818			

Dissatisfaction with the nostril size negatively influenced willingness for surgery (estimate = -0.954, p = 0.009; 95% CI = -1.674 to -0.234). This suggests that for some individuals, concerns about nostril size may not translate into a desire for surgery, or other factors might outweigh this specific aesthetic concern in their decision-making. Conversely, neutrality, satisfaction, or strong dissatisfaction with nostril size did not show a statistically significant effect on willingness when compared to the reference category “Strongly Satisfied.”

Similarly, dissatisfaction with the nasal height reduced the willingness for rhinoplasty significantly. The estimated coefficient for dissatisfaction with nasal height was negative (estimate = -0.837, p = 0.042; 95% CI = -1.645 to -0.029). This finding parallels the observation of nostril size, suggesting a nuanced relationship where specific aesthetic dissatisfaction may not always align with a higher propensity for surgical correction. Again, other levels of satisfaction with nasal height (neutral, satisfied, strongly dissatisfied) did not reach statistical significance in predicting willingness.

Respondents who were dissatisfied with the overall shape of their nose were more open to undergoing surgery (estimate = 1.947, p < 0.001; 95% CI = 1.129 to 2.764). This underscores the importance of nasal harmony and aesthetics in deciding on rhinoplasty.

A strong dissatisfaction with the general shape of the nose increased the willingness to undergo rhinoplasty (estimate = 3.041, p < 0.001; 95% CI = 1.847 to 4.234). This finding suggests that profound dissatisfaction with one’s overall nasal appearance is a significant motivator for seeking surgical correction (Table 1).

In other satisfaction categories (neutral, satisfied, or strongly dissatisfied with nostril size, neutral, satisfied, or strongly dissatisfied with nasal height, and neutral or satisfied with general nasal shape), the effects were not statistically significant. The reference category for all satisfaction questions was “Strongly Satisfied,” meaning the estimates represent the change in log-odds of willingness to undergo rhinoplasty relative to being strongly satisfied with that particular nasal feature.

Gender differences in nasal satisfaction

To assess whether satisfaction levels with various nasal features differed between genders, independent samples t-tests were performed (Table 3). The results consistently demonstrated significant gender disparities across numerous aesthetic aspects of the nose.

**Table 3:** Relationship between nasal satisfaction and gender.

	Levene’s test for equality of variances			
	F	Significance	t	df
What is your satisfaction with the function of the nose, such as breathing and sense of smell?	0.091	0.763	0.089	729
	-	-	0.089	708.527
How satisfied are you with the shape of the nose tip?	28.280	<0.001	-5.069	729
	-	-	-5.015	671.553
How satisfied are you with the shape of the alae of the nose?	19.263	<0.001	-4.144	729
	-	-	-4.109	683.471
What is your satisfaction with the shape of the nasal bridge?	12.538	<0.001	-3.460	729
	-	-	-3.437	691.096
What is your satisfaction with the size of the nostril?	23.932	<0.001	-3.656	729
	-	-	-3.621	677.559
What is your satisfaction with the color of the skin on the nose?	0.169	0.681	0.326	729
	-	-	0.327	718.819
What is your satisfaction with the height of the nose?	10.697	0.001	-3.698	729
	-	-	-3.676	695.220
What is your satisfaction with the shape of the nose in general?	25.624	<0.001	-5.806	729

Statistically significant gender differences were identified in satisfaction with the shape of the nose tip (F = 28.280, p < 0.001), the shape of the alae of the nose (F = 19.263, p < 0.001), the shape of the nasal bridge (F = 12.538, p < 0.001), the size of the nostrils (F = 23.932, p < 0.001), the height of the nose (F = 10.697, p = 0.001), and the general shape of the nose (F = 25.624, p < 0.001). In all instances with significant differences, males reported higher satisfaction than females. The negative mean differences from t-tests show females rated their satisfaction lower for these nasal features.

Conversely, no statistically significant gender differences were found in satisfaction with the nasal functional aspects, such as breathing and sense of smell (F = 0.091, p = 0.763). Similarly, satisfaction with the color of the skin of the nose also showed no significant gender difference (F = 0.169, p = 0.681). This suggests that while aesthetic perceptions of the nose vary significantly between genders, functional aspects are perceived more similarly. This aligns with literature indicating women often have higher body image dissatisfaction and seek aesthetic improvements.

Age group differences in nasal satisfaction

A one-way ANOVA was conducted for various aspects of nasal satisfaction (Table 4). The key purpose of the ANOVA was to determine whether there were statistically significant differences between and within age groups in their satisfaction ratings. The results indicate that the age group was not a significant factor influencing satisfaction ratings for any of the analyzed nasal characteristics.

**Table 4:** Relationship between nasal satisfaction and age groups.

Analysis of variance						
		Sum of squares	df	Mean square	F	Significance
What is your satisfaction with the function of the nose, such as breathing and sense of smell?	Between groups	7.323	3	2.441	1.810	0.144
	Within groups	980.310	727	1.348	-	-
	Total	987.633	730	-	-	-
How satisfied are you with the shape of the nose tip?	Between groups	6.429	3	2.143	1.157	0.325
	Within groups	1,346.816	727	1.853	-	-
	Total	1,353.245	730	-	-	-
How satisfied are you with the shape of the alae of the nose?	Between groups	2.899	3	0.966	0.506	0.679
	Within groups	1,389.266	727	1.911	-	-
	Total	1,392.164	730	-	-	-
What is your satisfaction with the shape of the nasal bridge?	Between groups	5.906	3	1.969	1.077	0.358
	Within groups	1,328.953	727	1.828	-	-
	Total	1,334.859	730	-	-	-
What is your satisfaction with the size of the nostril?	Between groups	7.879	3	2.626	1.352	0.256
	Within groups	1,412.442	727	1.943	-	-
	Total	1,420.320	730	-	-	-
What is your satisfaction with the color of the skin on the nose?	Between groups	0.210	3	0.070	0.053	0.984
	Within groups	969.639	727	1.334	-	-
	Total	969.850	730	-	-	-
What is your satisfaction with the height of the nose?	Between groups	6.385	3	2.128	1.120	0.340
	Within groups	1,381.936	727	1.901	-	-
	Total	1,388.320	730	-	-	-
What is your satisfaction with the shape of the nose in general?	Between groups	5.459	3	1.820	0.981	0.401
	Within groups	1,348.706	727	1.855	-	-
	Total	1,354.164	730	-	-	-

There were no statistically significant differences across age groups for satisfaction with the function of the nose, including breathing and sense of smell (F = 1.810, p = 0.144). Similarly, there were no significant differences in satisfaction with the shape of the nose tip (F = 1.157, p = 0.325), the shape of the alae of the nose (F = 0.506, p = 0.679), the shape of the nasal bridge (F = 1.077, p = 0.358), the size of the nostrils (F = 1.352, p = 0.256), the color of the skin of the nose (F = 0.053, p = 0.984), the height of the nose (F = 1.120, p = 0.340), and the general shape of the nose (F = 0.981, p = 0.401). The consistency of p-values being greater than 0.05 across all satisfaction aspects indicates that satisfaction with one’s nose does not vary significantly across age groups included in this study (Table 4).

Age and motivations for rhinoplasty

Analysis using Pearson’s chi-square test indicated no statistically significant relationship between age and the motivations for undergoing rhinoplasty (p = 0.112, chi-square value = 263.752, df = 237). This suggests that reasons for rhinoplasty, aesthetic or functional, do not vary significantly with age.

Gender and motivations for rhinoplasty

The relationship between gender and motivations for rhinoplasty was nuanced. Pearson’s chi-square test demonstrated a statistically insignificant difference between gender and motivations for rhinoplasty at the conventional 0.05 level (p = 0.064, chi-square value = 98.958, df = 79). Men and women in this study population do not significantly differ in their primary motivations for seeking rhinoplasty. However, the likelihood ratio test indicated a significant relationship (p < 0.001, chi-square value = 130.307, df = 79). This discrepancy between the Pearson’s chi-square and likelihood ratio tests suggests a weak or complex relationship that may not be captured by the Pearson statistic, especially with low expected counts, as noted in this analysis (95.0% had an expected count of less than 5). Further qualitative or advanced statistical analysis is needed to understand the complex relationship between gender and motivations for rhinoplasty (Table 5).

**Table 5:** Motivation for Rhinoplasty considering various factors (age, gender). ^a^: 152 cells (95.0%) have an expected count of less than 5. The minimum expected count is 0.47.

Age			
Chi-square tests			
	Value	df	Asymptotic significance (two-sided)
Pearson chi-square	263.752^a^	237	0.112
Likelihood ratio	155.525	237	1.000
Number of valid cases	731	-	-
Gender			
Chi-square tests			
	Value	df	Asymptotic significance (two-sided)
Pearson chi-square	98.958^a^	79	0.064
Likelihood ratio	130.307	79	<0.001
Number of valid cases	731	-	-

Educational level and motivations for rhinoplasty

The analysis of educational level and motivations for rhinoplasty revealed a statistically significant relationship. Pearson’s chi-square test yielded a p-value of less than 0.001 (chi-square value = 684.941, df = 395). This suggests educational attainment influences motivations for rhinoplasty, reflecting awareness. Further investigation may identify which educational levels prioritize aesthetic or functional concerns. Similar to the gender analysis, many cells had low expected counts, which affects the strength of interpretation of the association of these results.

Employment status and motivations for rhinoplasty

Pearson’s chi-square test indicated no statistically significant relationship with motivations for rhinoplasty (p = 0.103, chi-square value = 180.890, df = 158). This indicates an insignificant prediction whether they seek rhinoplasty for aesthetic or functional reasons. Similar to other analyses, the low expected counts (96.7% had an expected count of less than 5) may affect how these findings are interpreted. The likelihood ratio test for employment status was significant (p = 0.019). The non-significant Pearson’s chi-square test suggests caution in interpreting a strong direct relationship (Table 6).

**Table 6:** Motivation for rhinoplasty considering various factors (educational level, employment status). ^a^: 470 cells (97.9%) have an expected count of less than 5. The minimum expected count is 0.00;^ b^: 232 cells (96.7%) have an expected count of less than 5. The minimum expected count is 0.25.

Educational level			
Chi-square tests			
	Value	df	Asymptotic significance (two-sided)
Pearson chi-square	684.941^a^	395	<0.001
Likelihood ratio	203.064	395	1.000
Number of valid cases	731	-	-
Employment status			
Chi-square tests			
	Value	df	Asymptotic significance (two-sided)
Pearson chi-square	180.890^b^	158	0.103
Likelihood ratio	197.155	158	0.019
Number of valid cases	731	-	-

## Discussion

The study provides valuable insights into the complex factors predicting willingness to undergo rhinoplasty. Four significant predictors were identified, including the relationship between dissatisfaction with the general nasal shape and surgical interest. Strong dissatisfaction with overall nasal shape proved to be the most powerful predictor (estimate = 3.041, p < 0.001), suggesting that global perception of the nose influences decision-making more than specific anatomical features. This aligns with psychological research suggesting that holistic perceptions often supersede feature-specific concerns when individuals evaluate their appearance [14,15]. The substantial effect indicates that a strong general dissatisfaction may represent a distinct clinical population with more pronounced body image concerns.

In addition, dissatisfaction with nostril size (estimate = -0.954, p = 0.009) and nose height (estimate = -0.837, p = 0.042) were associated with decreased willingness to undergo surgery. This finding suggests that individuals with isolated concerns about these features might perceive them as minor issues that do not warrant surgery. In addition, awareness of surgical risks associated with modifying these features may lead to more conservative decision-making [16]. This highlights the intricate relationship between anatomical dissatisfaction and surgical motivation.

The comparison of genders revealed that females were significantly less satisfied than males with the tip of the nose, alae, bridge of the nose, size of the nostrils, height, and overall shape. The gender difference may reflect more widespread sociocultural trends in which females feel more judgment and pressure regarding their appearance [17,18]. However, the motivation for rhinoplasty was found to have only a marginal gender effect (p = 0.064), suggesting that while women may experience greater nasal dissatisfaction, translating this dissatisfaction into surgical intent involves additional mediating factors that warrant further investigation.

The insignificant age-related differences in nasal satisfaction and surgical motivation contradict some existing literature suggesting age-related trends in cosmetic surgery interest [19]. This may indicate that nasal aesthetics represent a relatively stable concern throughout life, unlike other appearance features that may become more prominent with age. Alternatively, the age grouping methodology may have obscured more subtle age-related patterns that could emerge with different analytical approaches.

A significant relationship was found between educational level and rhinoplasty motivations (p < 0.001). This strong association suggests that educational background may influence individual surgical goals, perhaps reflecting differences in health literacy, access to information, or sociocultural contexts associated with educational attainment [20-22]. Nevertheless, the nature of these educational differences in motivation is an important area for future research.

The absence of significant associations between rhinoplasty motivations and nationality or employment status suggests that these factors may have a minimal influence on surgical decision-making. This finding challenges assumptions about the socioeconomic determinants of interest in cosmetic surgery, suggesting that psychological and perceptual factors may have a greater influence on rhinoplasty decisions.

The study highlights important implications for clinical practice, patient education, and future research directions. For clinical practice, assessing patients’ perception of their overall nasal shape is crucial, as it is the strongest predictor of surgical willingness. Surgeons should develop standardized methods to evaluate specific anatomical concerns and patients’ overall satisfaction with their nasal appearance. Dissatisfaction with certain features decreased surgical willingness, requiring clinicians to explore patients’ understanding of surgical possibilities and limitations related to these features. Moreover, it is worth noting that self-reported questionnaires are susceptible to misinterpretation, miscomprehension, hidden agendas, bias, and limited participation.

The gender differences in nasal satisfaction without corresponding differences in surgical motivation suggest a need for gender-sensitive approaches to patient counseling that address the potentially different pathways from dissatisfaction to surgical decision-making in men and women. Educational differences in rhinoplasty motivations indicate that patient communication should be tailored to diverse educational backgrounds, with particular attention to ensuring that all patients receive accessible information about both aesthetic and functional aspects of rhinoplasty, regardless of educational attainment.

Future research should explore decision-making among individuals who are highly dissatisfied with specific nasal features but hesitant about undergoing surgery. Additionally, longitudinal research is essential to understand how nasal satisfaction and surgical motivations evolve to identify critical periods for intervention. Developing and validating more nuanced assessment tools would help capture the complex relationship between specific anatomical concerns and global nasal perception. Furthermore, cross-cultural studies are critical to examining how sociocultural contexts influence the relationship between nasal dissatisfaction and surgical decision-making. Investigations into the potential mediating role of psychological factors, such as body image, self-esteem, and social anxiety, are also essential to understanding rhinoplasty decision pathways.

The in-depth study of individual nasal attributes and population variables provides a multidimensional understanding of rhinoplasty decision-making. The model’s fit is good (p < 0.001), indicating that the predictive model successfully explains the significant variability in surgical willingness, including both aesthetic and functional concerns.

Nonetheless, the study’s cross-sectional design limits causation or temporal association between surgical willingness and nasal dissatisfaction. Self-reported surgical willingness and nasal satisfaction could be influenced by social desirability or limited self-awareness. Lastly, the research focused on direct associations between the variables, and more complex mediating or moderating processes likely impact the relationship between surgical decision-making and nasal satisfaction.

## Conclusions

This study explores the complex relationship between predictors of surgical willingness for rhinoplasty. The main predictor is dissatisfaction with the overall nose shape. However, dissatisfaction with specific nose features, such as nose size and nostrils, was negatively correlated with surgical willingness. Females reported lower satisfaction in various aspects of the nose than males. Educational level is also positively associated with rhinoplasty motives. The decision regarding rhinoplasty depends on physical, psychological, and social factors. As cosmetic surgery becomes more accessible and socially accepted, patient variables such as perception, motivation, and concerns should be prioritized over fixed assumptions regarding specific features of the nose or demographic variables.

## Appendices

Gender satisfaction regarding their nose shape and their willingness to undergo rhinoplasty among the Al-Madinah population, Saudi Arabia, 2021-2022

Dear population of Madina,

We put in your hand this questionnaire, which is a part of research aimed at studying the gender satisfaction regarding the nose shape and the willingness to undergo rhinoplasty among the population of Al-Madinah. We would like you to know that participation in this research is completely voluntary, and the collected data will be used only for scientific purposes, with full privacy and confidentiality.

Thank you for your cooperation.

For any questions, you can contact us at: nisreenalbouq@gmail.com

1. I agree to participate in this research voluntarily and of my own will

- I agree

- I don’t agree

Demographic data

2. Age

- 18-28

- 29-39

- 40-50

- Above 50

3. Gender

- Male

- Female

4. Nationality

- Saudi

- Non- Saudi

5. Educational level

- Illiterate

- Primary school

- Intermediate school

- Secondary school

- graduate degree

- Post-graduate degree

6. Job

- Student

- Employed

- Non-employed

Assessing the participant’s satisfaction regarding their nose shape and appearance:

- Very satisfied = 1 satisfied = 2 moderately = 3 dissatisfied = 4 Very dissatisfied = 5

7. How satisfied are you with your total nasal functioning? 1-5

8. How satisfied are you with your nasal tip appearance? 1-5

9. How satisfied are you with your nasal wing(s) appearance? 1-5

10. How satisfied are you with your nasal dorsum appearance? 1-5

11. How satisfied are you with the size of your nostril(s)? 1-5

12. How satisfied are you with the color of your nasal skin? 1-5

13. How satisfied are you with your nasal tip position? 1-5

14. How satisfied are you with your total nasal appearance? 1-5

15. Do you think about doing rhinoplasty?

- Yes

- No

- Maybe

16. Why do you want to do rhinoplasty?

- The appearance of my nose makes me feel embarrassed and insecure

- It hinders my ability to communicate with people

- Because of people’s comments on the appearance of my nose

- Because of the influence of social media

- I don't want to have a rhinoplasty

- Others:
